# Predicting the potential global distribution of diosgenin-contained *Dioscorea* species

**DOI:** 10.1186/s13020-018-0215-8

**Published:** 2018-11-19

**Authors:** Liang Shen, Jiang Xu, Lu Luo, Haoyu Hu, Xiangxiao Meng, Xiwen Li, Shilin Chen

**Affiliations:** Institute of Chinese Materia Medica, China Academy of Chinese Medical Sciences, Beijing, 100700 China

**Keywords:** *Dioscorea* species, Diosgenin material, GMPGIS, Potentially suitable areas, Climate characteristics

## Abstract

**Background:**

Diosgenin, mainly extracted from wild diosgenin-contained *Dioscorea* species, is a well-known starting material of steroidal and contraceptive drugs. However, due to large market demand and increasingly ecological damage, wild *Dioscorea* species resources available have been gradually declining. Therefore, identification of new potential ecological distribution of diosgenin-contained *Dioscorea* species is necessary for diosgenin production.

**Methods:**

In this study, a large occurrence dataset (1808 data points) of diosgenin-contained *Dioscorea* species was obtained from Eastern Asia, Southern North America and Southern Africa. Along with the data for six critical environmental parameters and one soil factor, Geographic Information System for Global Medicinal Plant was applied to predict the potential suitable distribution of *Dioscorea* species.

**Results:**

The results showed that the potential distribution of these *Dioscorea* species covered a wide field, and that new ecological suitability areas were mainly distributed in the central region of South America, the southern part of the European and coastal region of Oceania. Jackknife test indicated that annual precipitation and annual mean radiation were the important climatic factors controlling the distribution of *Dioscorea* species.

**Conclusions:**

The suitable areas and critical climatic factors will serve as a useful guide for diosgenin-contained *Dioscorea* species conservation and cultivation in ecological suitable areas.

**Electronic supplementary material:**

The online version of this article (10.1186/s13020-018-0215-8) contains supplementary material, which is available to authorized users.

## Background

Diosgenin is a versatile starting material for the manufacture of steroidal drugs, and it is mainly extracted from *Dioscorea* species [1]. Steroidal has strong anti-infection, anti-allergic and other pharmacological effects which plays an important role in the treatment of rheumatoid arthritis, heart disease, peptic ulcer disease, etc. [2]. Diosgenin has also been prescribed as an oral contraceptive with large market demands in recent years. There are 137 kinds of *Dioscorea* species containing diosgenin, 41 kinds of which contain over 1% diosgenin with great utilization value [3]. However, their resources have been declining quickly due to excessive harvesting, and some species are even getting nearly extinct. Nevertheless *Dioscorea* became a major source to produce steroid hormone due to the failure of accomplishing chemical synthesis of steroids [4]. In India, approximate 100% production of steroidal drugs is based on diosgenin material from *Dioscorea* species [5]. Therefore, it is necessarily needed to explore approaches in conservation and cultivation of diosgenin-contained *Dioscorea* species to obtain diosgenin materials.

Recently, booming market demands boosted the expansion of introduction and cultivation of *Dioscorea* species worldwide. China and Mexico are the two main production countries, which account for 67% of diosgenin yield with the richest *Dioscorea* resource in the world [1]. However, the yield and quality of diosgenin was declined due to the lack of high-quality germplasm, unknown suitable plant region and shortage of useful technology [6]. As far as we are concerned, there still exist some *Dioscorea* species cultivated in rural China, what remains confusing to us is that whether they can be used as diosgenin source or not has not been testified yet [7]. Understanding the requirements of habitat conditions of these *Dioscorea* species may be useful for managing population recovery and plantation, as well as promoting economic growth. The cultivation methods for *Dioscorea* species, such as breeding, management, and planting have been discussed by previous reports [8, 9]. Nevertheless, suitable distribution and ecological requirements of these *Dioscorea* species remain unknown. Quite a limited number of studies have assessed the distribution patterns of the *Dioscorea* species, and there was an article about habitats across Bangladesh by the species distribution modeling (SDM) [10]. Additionally, the *D. nipponica* potential distribution was assessed across Jilin province in China by the MaxEnt. High fitness suitable areas were also identified to concentrate at the central and southern regions of Jilin [11]. Hence, it is essential to conduct conservation and cultivation study on a global scale for *Dioscorea* species which analyzes ecological factor similarities include climate, soil between the origin and introduction sites and to draw an accurate global cultivation region map.

With the development of network technology, the geographic information system (GIS) is just an ideally digital mapping tool adopted for geospatial database creation, data integration and modeling [12]. GMPGIS can predict the impact of climate on medicinal plants potential distribution model, and the model is verified successfully in predicting the distribution of *Panax* species [13–15]. It is of great significance to predict the potential suitable distribution of *Dioscorea* species by GMPGIS with primary ecological factors for their protection and utilization. In this study, we analyzed the potential global suitable habitats of diosgenin-contained *Dioscorea* species by means of GMPGIS based on six climate variables and soil factor, and mapped the key environmental variables that constrain the geographical distribution of those *Dioscorea* species by Jackknife test. These results will provide a valuable reference for conservation, introduction and cultivation of diosgenin-contained *Dioscorea* species worldwide.

## Materials and methods

### Species data

**Fig. 1:**
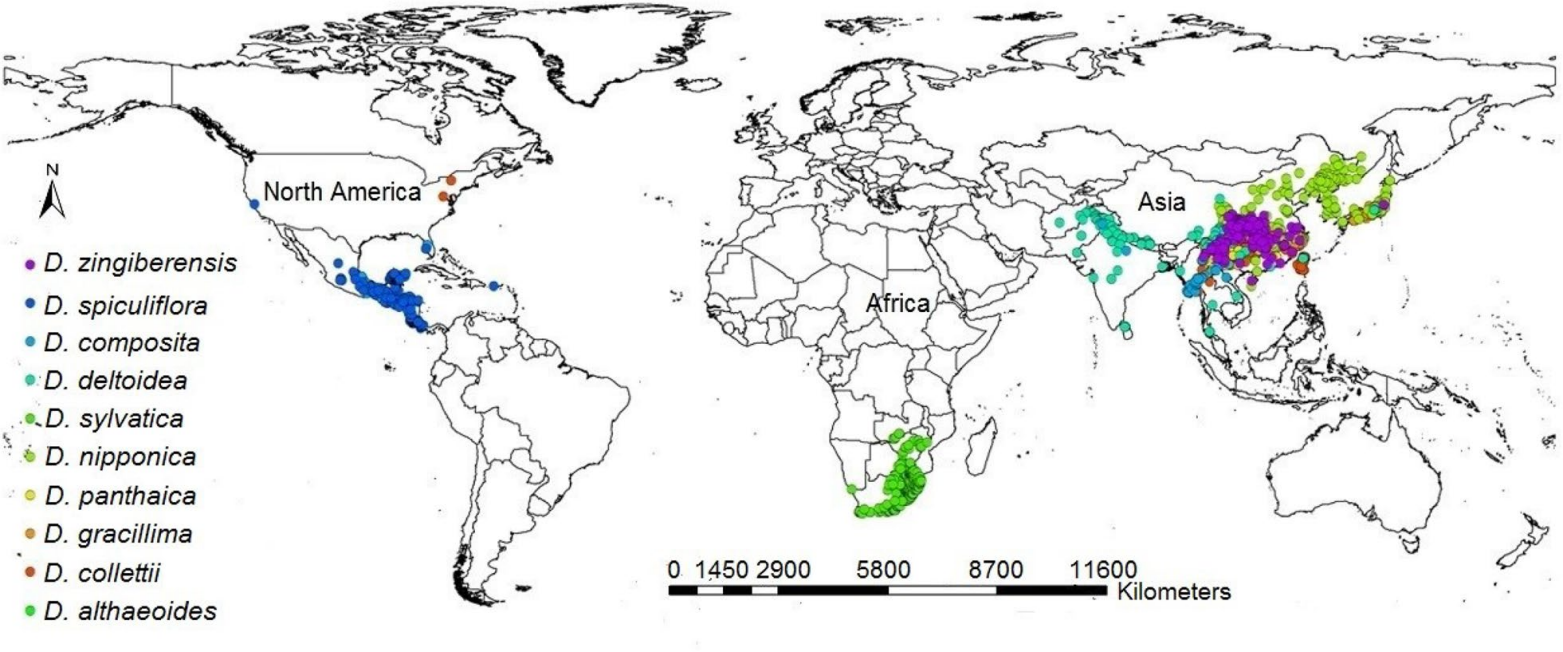
Global spatial distribution of occurrence records of diosgenin-contained *Dioscorea* species around the world. The map was plotted using ArcGIS 10.2 (http://www.esri.com/)

In this study, ten *Dioscorea* species were selected in accordance with the principle of higher diosgenin content, crop yield and industrialized application [4, 16–18]. Samples points of *Dioscorea* species were drawn from main producing areas, wild distribution and historical growing region [13]. Data on the distribution of *Dioscorea* species were obtained from the following sources: (1) the Global Biodiversity Information Facility Data Portal (GBIF, http://www.gbif.org/); (2) Royal Botanic Gardens, Kew (Kew, http://www.kew.org/); (3) the Chinese Virtual Herbarium (CVH: http://www.cvh.org.cn/); (4) relevant literature and field investigation. Additionally, sampling bias were reduced with regard to environmental conditions, only one sample was kept when replicated. Each sampling site was converted into geographic coordinates (World Geodetic System 1984 data) by ArcGIS (ver. 10.2) (http://www.esri.com/). Finally, a total of 1808 points were valid, and the samples points were mainly from China, Mexico, United States and South Africa, etc. (Fig. 1; Additional file 1: Table S1) [16–26].

### Environmental variables

Following the main controlling factors of the distribution and characteristics of medicinal plants, the prediction of model selection of variables should reflect the coldest and warmest temperatures, moisture, radiation and precipitation of species, and the most influential variables associated with diosgenin yield are considered as well [15]. In this study, the selection of mainly used variables was based on the biological characteristics of medicinal plants, references and the data analysis [13–15, 27, 28]. A total of six related ecological factors for medicinal plants were selected and down from the Worldclim database (http://www.worldclim.org/) (Period 1970–2000) (Additional file 1: Table S2) [29, 30], with a resolution of 2.5 arcmin-seconds, and availability of data of 10 *Dioscorea* species were in supply files (Additional file 2). The soil variables were obtained from Harmonized World Soil Database (http://www.iiasa.ac.at/). For region measurements, the layers were projected into UTM coordinates with the original data in WGS84. Global administrative areas come from the GADM database, and the version is 2.8 (http://www.gadm.org/).

### Species distribution modeling

**Fig. 2:**
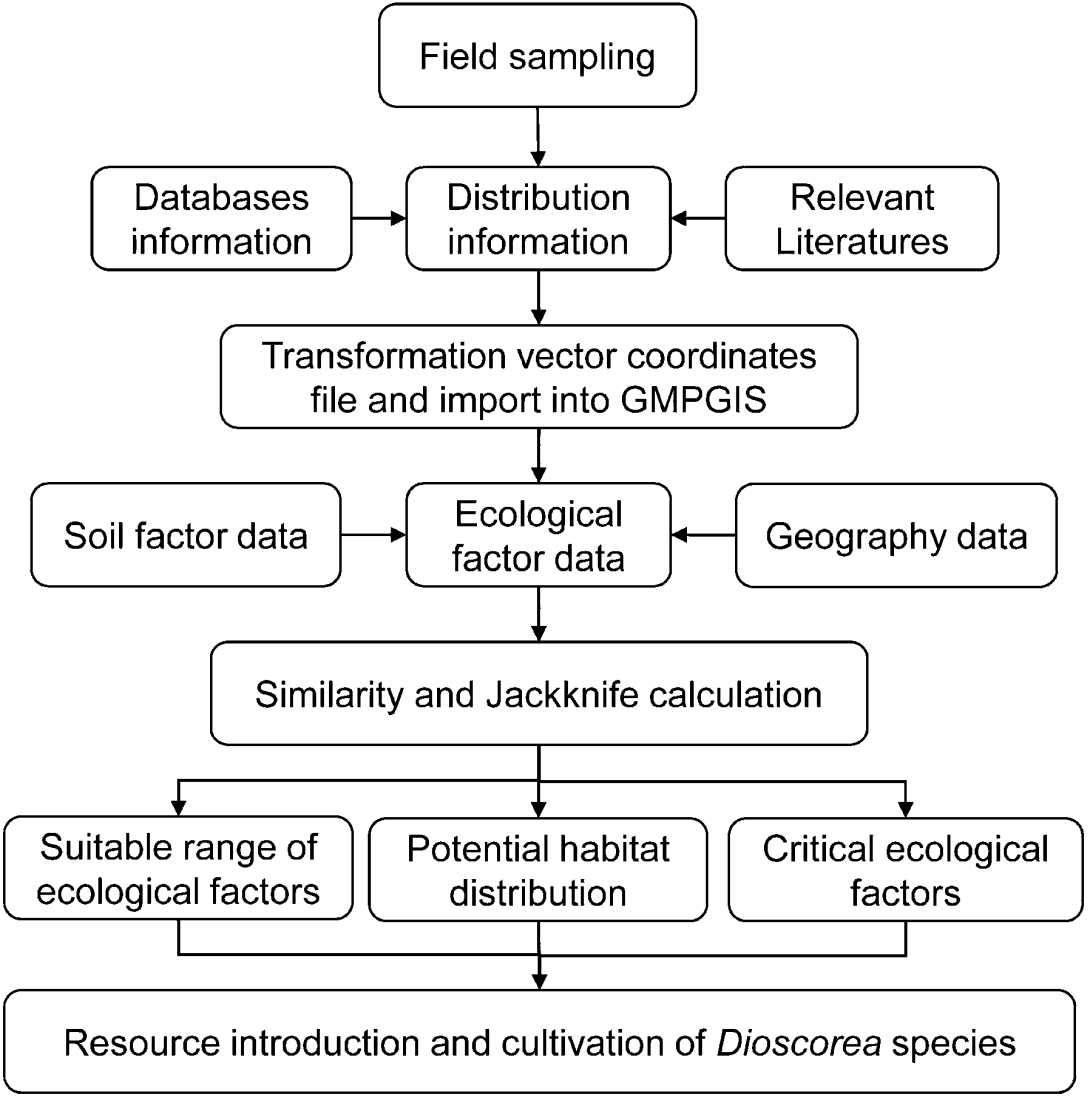
Flow-chart of the ecological suitability analysis for diosgenin-contained *Dioscorea* species

GMPGIS was a model using global geographic information system for medicinal plant distribution prediction, and it was self-developed by the Institute of Chinese Materia Medica, China Academy of Chinese Medical Sciences (CACMS) based on GIS technology. GMPGIS climate database was adopted from the World Clim-Global Climate Data [29] and CliMond (https://www.climond.org/), and the soil database was obtained from Harmonized World Soil Database (HWSD) [13–15]. In GMPGIS, the occurrences of plant species with known distributions are related to climate data by using improved k-means method in Euclidean distances algorithms, and the accuracy of GMPGIS model has been successfully verified by six *Panax* plants [14, 15]. A suitable habitat map for *Dioscorea* species was established according to the following four main steps: linear normalization (a), grid-based spatial clustering, vector-based overlaying and suitable region analysis (b and c). The suitable soil layer and climatic factors in the Euclidean distance layer were intersected, and the predicted map was drawn [13, 15] (Fig. 2). A natural probabilistic explanation representing degrees of ecology suitability (0 = unsuitable to 0.999 = best habitat) was presented by the model logistic outcomes [31].

a $$v^{\prime}_{i} = \frac{{v_{i} - min_{A} }}{{max_{A} - min_{A} }} \times 100,$$

b $$E = \sum\limits_{i = 1}^{k} {\sum\limits_{{p \in D_{i} }} {dist\left( {p,d_{i} } \right)^{2} } } ,$$

c $$dist\left( {p,d_{i} } \right) = {\text{IF}}\left[ {min \le v_{i} \le max,\,0,\,\hbox{min} \left( {\left| {v^{\prime}_{i} - newmin_{A} } \right|,\,\left| {v^{\prime}_{i} - newmax_{A} } \right|} \right)} \right].$$

where (*vi*), the *vi* of *A* to *vi* in the range [*newminA*, *newmaxA*], and *di* is the scope of cluster *Di*, *newminA* is the minimum value after normalizing the layer, *newmaxA* is the maximum value after normalizing the layer.

In this research, analysis of the key environmental variables which constrained the geographical distribution of *Dioscorea* species were conducted by jackknife testing in MaxEnt version 3.3.3 k [32]. Parameter setting of modeling was as follows: The training set was 75% of the sampling data points. For the test set, 25% residual was used to examine the predictive ability of the MaxEnt, and the jackknife was used to test the weight. In order to prevent over-fitting of the test data, the regularization multiplier value was set as 1, and convergence threshold 0.00001 [33]. All other settings were kept as default value and output format settings [34].

### Phylogenetic relationship among *Dioscorea* species

The chloroplast genomes *matK* and *rbcL* sequence possessed highly interspecific differences and were capable of distinguishing medicinal plants at the species level [35]. In the present study, *matK* and *rbcL* sequences of ten diosgenin of *Dioscorea* species were downloaded from the GenBank database. Consensus sequences and coting generation were obtained by the software CondonCode Aligner V3.7.1 (CodonCode Co., USA). The sequences of *Dioscorea* species were aligned by Muscle, and the genetic distance was computed with MEGA6.0 software (http://www.megasoftware.net) by using K2P model [36]. A phylogenetic tree based on *matK* and *rbcL* was constructed by employing the neighbor-joining (NJ) tree method, and bootstrap tests were calculated with 1000 resamples to assess the statistical confidence in phylogenetic analysis. Accordance with the phylogenetically related genetic information inferred from APGIV, and same sequence of *Tacca chantieri* Andre and *Alisma plantago-aquatica* Linn downloaded from GenBank were chosen as our group when the NJ tree was built [37–39] (Additional file 1: Table S3). The Minimum Standards of Reporting Checklist contains details of the experimental design, and statistics, and resources used in this study (Additional file 3).

## Results

### Model performance and contribution of environmental variables

*D. deltoidea* and *D. nipponica* showed a significantly different performance in regions and climatic factors comparing to all the other species (Table 1, Additional file 4: Figure S1). *D. deltoidea* and *D. composita* variation of climatic factors were the maximum and minimum, respectively. In six climatic factors, the maximal variation factor was T-warm, while the minimal change factor was T-cold. Soil types of ten *Dioscorea* species were mainly in Acrisols, Alisols, Andosols, Anthrosols, Cambisols, Fluvisols, and so on. Thus, the results indicated that these ecological conditions were optimal for the growth of diosgenin-contained *Dioscorea* species.

**Table 1:** Range values (minimum–maximum) of the ecological factors for ten diosgenin-contained *Dioscorea* species

Species	T-aver (°C)	T-warm (°C)	T-cold (°C)	Precipitation (mm)	Radiation (W m^−2^)	Humidity (%)
*D. althaeoides*	4.80 to 21.50	12.00 to 28.50	− 4.10 to 14.20	347 to 1736	125.58 to 153.30	41.90 to 76.40
	Soil types: Acrisols, Alisols, Andosols, Anthrosols, Chernozems etc.					
*D. collettii*	4.40 to 27.20	10.90 to 29.30	− 4.10 to 26.20	543 to 4854	119.08 to 168.05	50.20 to 77.00
	Soil types: Acrisols, Alisols, Anthrosols, Cambisols, Fluvisols etc.					
*D. composita*	12.40 to 27.20	13.30 to 32.80	4.80 to 25.20	785 to 4143	136.80 to 198.97	51.60 to 78.10
	Soil types: Acrisols, Andosols, Arenosols, Cambisols, Fluvisols etc.					
*D. deltoidea*	− 6.70 to 28.10	4.20 to 34.10	− 18.20 to 26.1	142 to 3774	122.83 to 228.01	38.40 to 76.50
	Soil types: Acrisols, Andosols, Arenosols, Cambisols, Fluvisols etc.					
*D. gracillima*	2.00 to 19.50	13.20 to 28.70	− 18.70 to 10.3	543 to 2821	116.73 to 144.76	56.80 to 76.40
	Soil types: Acrisols, Alisols, Andosols, Anthrosols, Cambisols etc.					
*D. nipponica*	− 1.70 to 25.90	5.60 to 28.80	− 20.30 to 220	295 to 3338	113.89 to 165.42	47.60 to 76.80
	Soil types: Acrisols, Alisols, Andosols, Arenosols, Anthrosols etc.					
*D. panthaica*	6.20 to 20.30	13.20 to 27.50	− 3.50 to 15.10	543 to 1743	122.15 to 154.77	50.20 to 75.70
	Soil types: Acrisols, Alisols, Anthrosols, Cambisols, Fluvisols, etc.					
*D. spiculiflora*	11.20 to 27.10	13.10 to 29.30	9.00 to 26.30	344 to 4296	150.92 to 207.56	46.50 to 80.50
	Soil types: Acrisols, Andosols, Cambisols, Gleysols, Kastanozems etc.					
*D. sylvatica*	6.20 to 24.00	10.40 to 26.40	1.20 to 20.20	68 to 1600	161.28 to 206.69	46.00 to 73.80
	Soil types: Acrisols, Arenosols, Calcisols, Cambisols, Fluvisols etc.					
*D. zingiberensis*	7.40 to 24.20	16.60 to 28.70	− 3.50 to 18.80	543 to 1849	117.70 to 150.39	52.90 to 76.00
	Soil types: Acrisols, Alisols, Andosols, Anthrosols, Cambisols etc.					

*T-aver* annual mean temperature, *T-warm* mean temperature of warmest quarter, *T-cold* mean temperature of coldest quarter, *Precipitation* annual precipitation, *Radiation* annual radiation, *Humidity* annual relative humidity

**Fig. 3:**
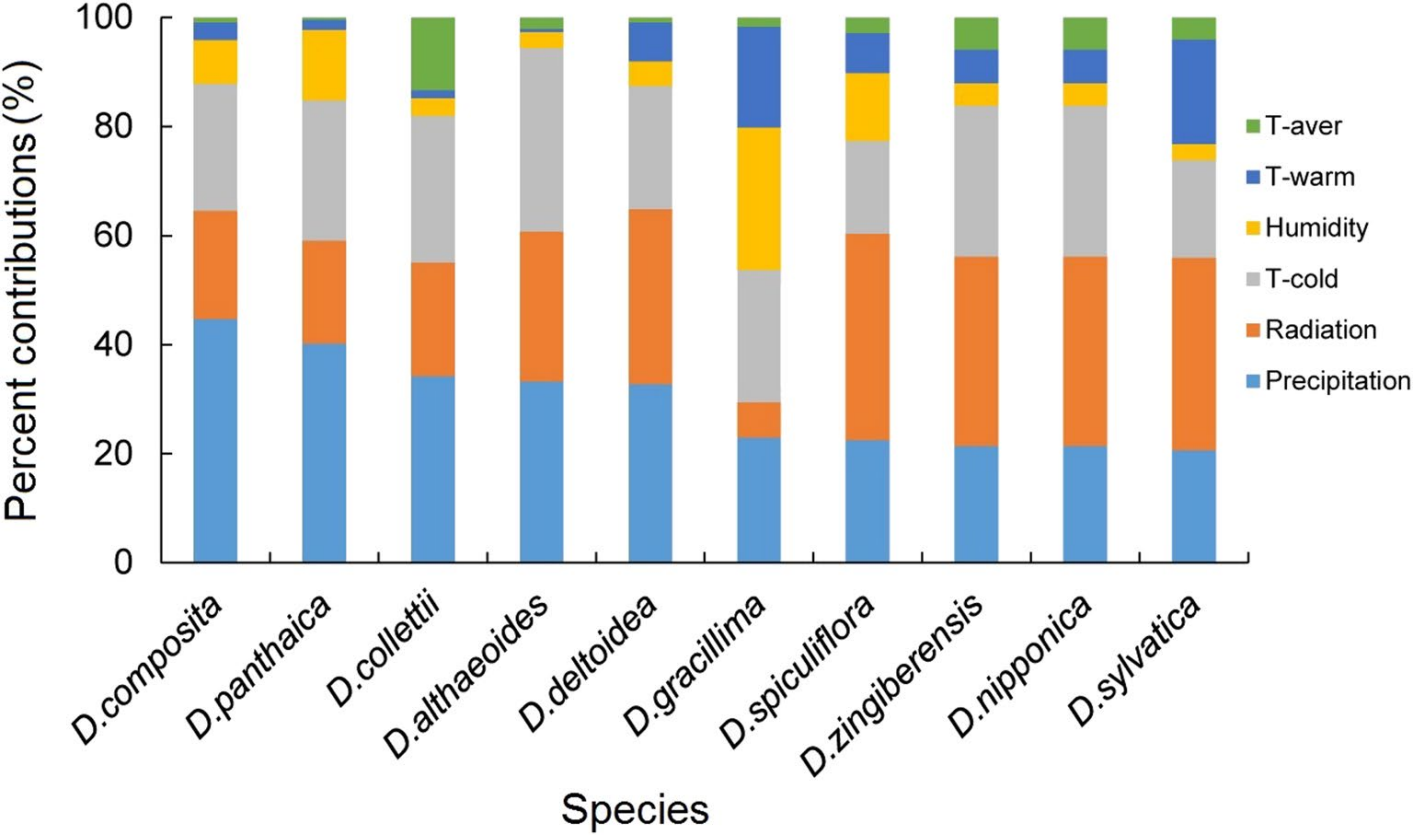
Contribution of individual variable to habitat suitability allowing the diosgenin-contained *Dioscorea* species

The contributions of each ecological factor were revealed by Jackknife test (Fig. 3). According to the result, annual precipitation and annual mean radiation were the key factors driving the modelled distribution of most of the ten *Dioscorea* species. For four of the taxa (*D. zingiberensis*, *D. sylvatica*, *D. spiculiflora* and *D. nipponica*), radiation emerged as an important contributor to the modelled distribution, as well as precipitation probability for other three taxa (*D. composita*, *D. deltoidea* and *D. panthaica*), humidity for *D. gracillima* and T-cold for *D. althaeoides*. The T-cold was also an important contributor to the modelled distribution in whole species, except for the *D. spiculiflora*, *D. deltoidea* and *D. sylvatica*. None of the species was strongly constrained by T-aver or T-warm. The range of suitable environmental factors provided a useful reference for the cultivation of those ten *Dioscorea* species.

### Potential global distribution

**Fig. 4:**
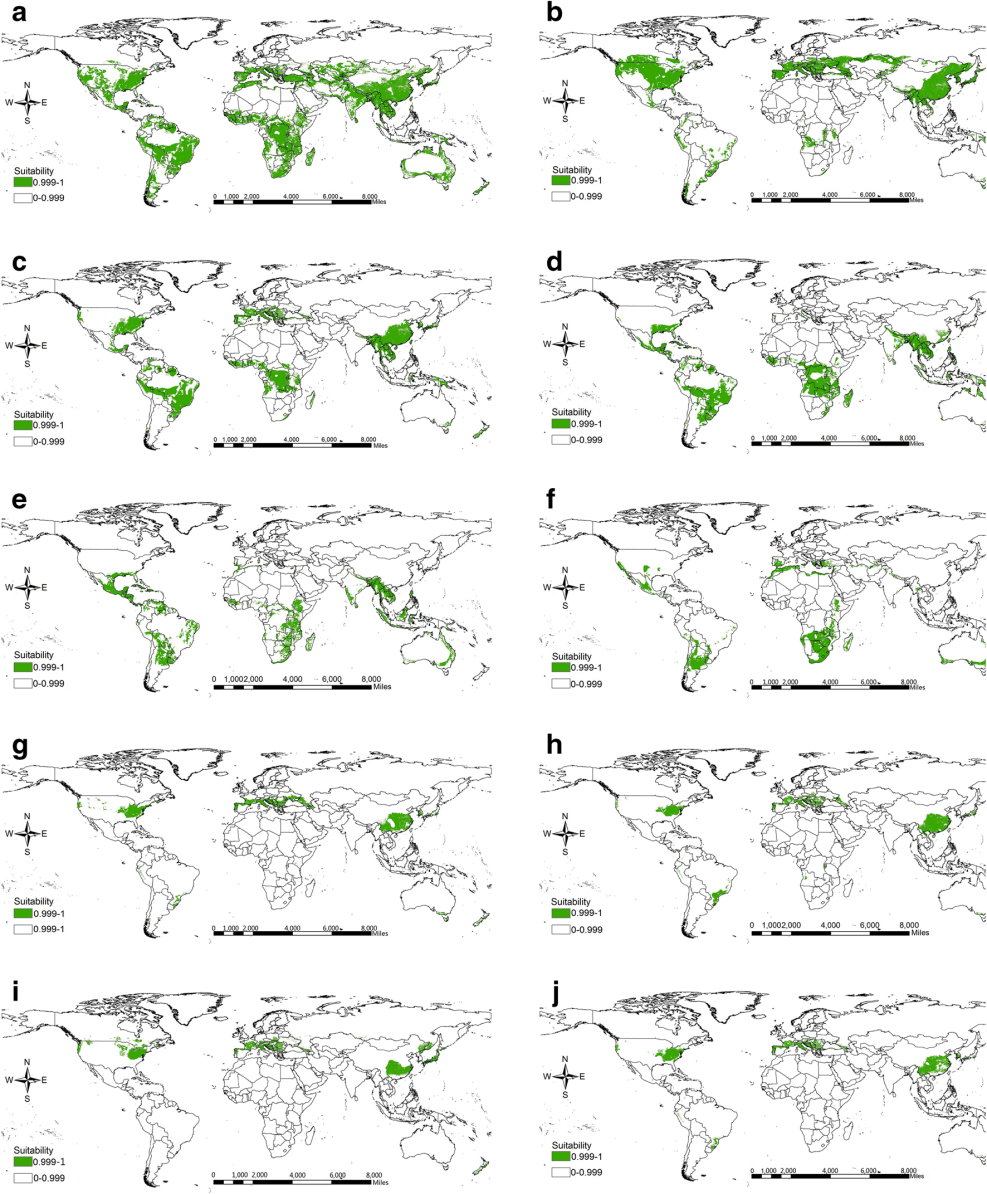
Maximum similarities of the (left side) ecological distribution and (right side) suitable areas for diosgenin-contained species from *Dioscorea.* **a** *D. deltoidea*, **b** *D. nipponica*, **c** *D. collettii*, **d** *D. composita*, **e** *D. spiculiflora*, **f** *D. sylvatica*, **g** *D. althaeoides*, **h** *D. zingiberensis*, **i** *D. gracillima*, **j** *D. panthaica*

**Fig. 5:**
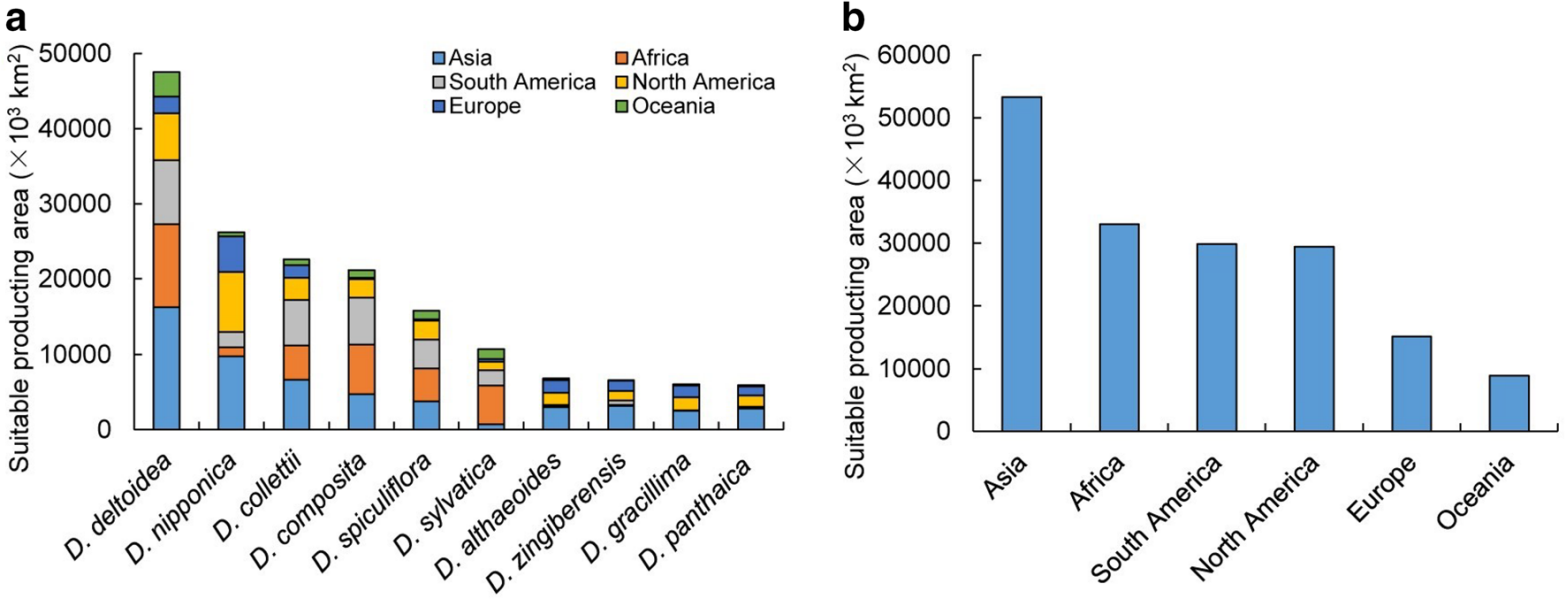
Results of diosgenin-contained plants of ten *Dioscorea* species for suitable regions area in continents worldwide based on GMPGIS. **a** Suitable producing area of species, **b** suitable producing area of each continent

According to ecological similarity growth of *Dioscorea* species, the results showed that the potential distribution areas of ten *Dioscorea* species stretched a rather wide area (Fig. 4, Additional file 1: Table S4). Ecological range of *D. deltoidea*, *D. collettii*, *D. composita* and *D. spiculiflora* covered a wide field, and the potential areas were more than (158.16–465.91) × 10^5^ km^2^, mainly distributed in the central region of South America, southern part of Africa and Asia (Fig. 4a, c–e). *D. nipponica* was potentially distributed in most parts of the earth, but mainly in most parts of North America, southern part of Europe and the eastern part of Asia, and suitable areas were 262.33 × 10^5^ km^2^ (Fig. 4b). *D. sylvatica* was mainly potentially distributed in southern part of Africa, South America and Asia, and potentially suitable areas are 107.40 × 10^5^ km^2^ (Fig. 4f). In contrast, the potential areas of *D. althaeoides*, *D. zingiberensis*, *D. gracillima* and *D. panthaica* areas were within the scope of (59.02–68.37) × 10^5^ km^2^, and mainly distributed in the eastern part of North America, southern part of European and Asia (Fig. 4g–j). Based on the area of producing district, there were some countries suitable for promoting planting such as China, Mexico, United States, Brazil, France, Japan, North Korea, Indonesia, India, Australia and so on (Additional file 4: Figure S2). The results indicated that *D. deltoidea*, *D. nipponica* and *D. collettii* were proper plantations in Eastern Asia; *D. deltoide*a, *D. composita* and *D. spiculiflora* were proper plantation in North America; the proper plantation species in Southern Africa contained *D. deltoidea* and *D. sylvatica*, and *D. spiculiflora* were suitable cultivation in North America (Fig. 5). The suitable areas in Southern Europe and Oceania seemed limited, and the proper species were *D. deltoidea* and *D. nipponica*. Asia was found to be the largest planting area, and Oceania, the smallest.

### Phylogenetic relationships among ten *Dioscorea* species

**Fig. 6:**
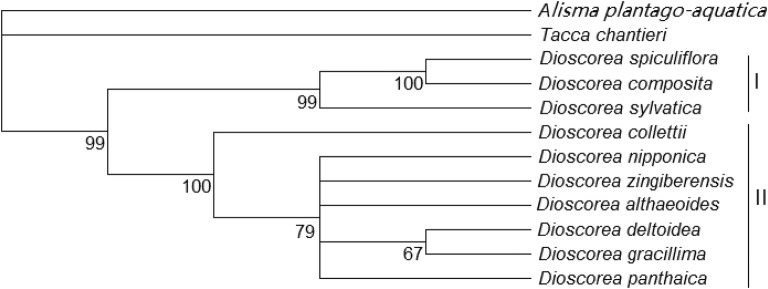
Neighbor-joining tree of the *matK* and *rbcL* genes from accessions belonged to *Dioscorea* Numbers at the nodes indicate bootstrap values (% over 1000 replicates). I (African and North America species) II (Asia species)

Phylogenetic trees of ten diosgenin-contained species from *Dioscorea* were created by the NJ method. The result of the analysis on the bootstrap values above 50% is given (Fig. 6). The tree was derived by alignment of concatenation *matK* and *rbcL* sequences. The moderately and strongly supported groups of phylogenetic trees were clearly shown to be two trees. Among ten *Dioscorea* species, two North America species of *D. spiculiflora* and *D. composita* and one African species *D. sylvatica* belonged to the same cluster (Cluster I), six Asian species such as *D. gracillima*, *D. althaeoides*, *D. panthaica*, *D. deltoidea*, *D. zingiberensis* and *D. nipponica* formed a moderate support to the same cluster (Cluster II). As expected, *D. collettii* and the six species above were in one group together. As can be evaluated from the branch length, the evolutionary divergence between two groups was significant, the outgroup were *Tacca chantieri* Andre and *Alisma plantago-aquatica* Linn.

## Discussion

In recent years, species distribution models (SDMs) developed to be a significant tool to estimate the impact of climate change on plant distribution [40, 41]. These models formed a correlation between species existence and its geophysical environment to predict the current potential distribution of species [42, 43]. GMPGIS is a model capable of indicating the range of ecological factors and the suitable potential ecological areas simply by running a relatively small number of present data, presenting accuracy in predicting suitable region of plants, such as *Panax ginseng*, *Panax japonicas* [14, 15]. In this study, sample points from 108 to 274 of ten higher diosgenin-contained *Dioscorea* species were used in GMPGIS, the model suggested that the potentially suitable areas of *Dioscorea* species shared similarity distribution with occurrence dataset of ten species [21, 22], and some unknown potential areas were predicted in our study. Those data reiterated the conclusion of GMPGIS, which indicated that it was a viable accuracy method for modelling plant distributions. Consequently, the combination of GMPGIS and jackknife test will play a dominate role in the prediction of suitable areas for medicinal species protection and cultivation.

Generally, environmental factors are the ones to blame for the driving forces of changes in *Dioscorea* species distribution and various ecological factors contribute to the growth of different species [15]. Most researches declared that *Dioscorea* species should be cultivated in high-temperature and fertile soil sandy loam, and its growth was determined by water shortage, strong light radiation and cold injury [44–46]. Therefore, summarizing the suitable climate factor of *Dioscorea* species distribution could provide a scientific basis for high-quality plants cultivation. This study found that the annual precipitation and annual mean radiation were the key factors driving the *Dioscorea* species distribution according to environment variable contributions. These results were confirmed by the results from current ecological feature study on *Dioscorea* species distribution. Seven *Dioscorea* species are distributed in Sichuan basin, China, and those plant species require a relatively damp habitat with low radiation to grow [45]. Previous studies on the relationship between effective constituent and ecological factors suggested that precipitation and radiation were the principal ecological factors affecting the diosgenin accumulated from *Dioscorea* species [47], and similarities or differences in growth habits could lead to different yields and qualities. Besides, the variety in annual mean radiation could also influence photosynthesis, and further affect the grow stage of diosgenin-contained *Dioscorea* species [48]. The results of this study proved a similar correlation between ecological factors and cultivation. In conclusion, during the process of introduction and cultivation of diosgenin plants, it is necessarily needed to make scientific plant management according to various suitable environmental conditions of *Dioscorea* species.

It seems fundamental to detect the potential distribution regions for the conservation and plantation of medicinal plants [49]. In this study, the modelled ecological niches and geographic distributions of these ten diosgenin-contained *Dioscorea* species showed a high degree of differentiation. The potential suitable areas of three Asia *Dioscorea* species (*D. deltoidea*, *D. nipponica* and *D. collettii*) were mainly located in the mid-east region of North America and South America, southern part of Africa and eastern part of Asia, and the potential global distributions were within the scope of (226.23–465.91) × 10^5^ km^2^. However, the other four Asia *Dioscorea* species (*D. zingiberensis*, *D. panthaica*, *D. althaeoides* and *D. gracillima*) were mainly potentially distributed in China, the United States and southern areas of Europe, and the potential suitable areas were within the scope of (59.02–68.37) × 10^5^ km^2^. It indicated that some of *Dioscorea* species were distributed within a very narrow region, whereas other taxa were widely distributed in the world. Perhaps genetic information is one of the most significant causes to determine the distribution of *Dioscorea* species. Consequently, to select “where to plant or grow” the primary thing to do before the introduction and cultivation of diosgenin plant.

Our model results indicated that diosgenin-contained *Dioscorea* species could be introduced to many undiscovered potential areas, such as south of Europe and central north of America, except for the original production regions of *Dioscorea* above. According to the diosgenin contents and potential distribution, *D. deltoidea* and *D. nipponica* were recommended plants in the Asia. *D. deltoidea* and *D. sylvatica* were suggested being planted in Africa, *D. composita* and *D. spiculiflora* were suitable to be cultivated in North America. Potential suitable regions prediction in this study could provide a scientific basis for *Dioscorea* species selection, as well as the introduction and cultivation worldwide. However, a field test is necessary before *Dioscorea* species cultivation in large areas, since the production would be affected by many other factors, such as local transport, natural disaster, and so on.

Phylogenetic trees of ten *Dioscorea* species were created by NJ methods. Among those species, seven from Asia formed a moderately supported group belonging to the same cluster. Three species of *D. spiculiflora*, *D. composita* and *D. sylvatica* belonged to another cluster, which came from Africa and North America. Previous studies showed that the Hengduan Mountains of China was a distributing center of *Dioscorea* species, and it also demonstrated a suitable potential area for *Dioscorea* species introduction and cultivation in this research [3]. Shen et al. reported seed traits character of *Haloxylon ammodendron* was strongly affected by climatic and geographical factors, and were moderately correlated with genetic diversity [50]. This study indicated that distribution region of species was correlated to its genetics and environment, and these potential suitable regions could introduce and cultivate *Dioscorea* species in the future.

Containing diosgenin, *Dioscorea* species owns a fine cultivated character and high reproduction, so the plantation scale of *Dioscorea* species in recent years has expanded constantly. However, cultivation distribution and ecological requirements of *Dioscorea* species remain chaotic and is in need of universal unification globally, yet confusion in its introduction and cultivation has led to a decline in yield and quality of diosgenin [7]. Our research result will provide a practical reference for the production of diosgenin in different areas worldwide. Combining with the research result, the plantation development directions of diosgenin-contained *Dioscorea* species in the future are (1) selecting suitable diosgenin species in accordance with the research results to conduct plantation; (2) strengthening ecological study on the quality of *Dioscorea* species, studying ecological characteristics of main cultivated species, and analyzing the influencing mechanism of environmental factors, such as light, temperature and water on the content and yield of diosgenin; (3) to develop a new variety of high-quality and stress-resistant *Dioscorea* species in the future.

## Conclusions

In this study, a large occurrence dataset of diosgenin-contained *Dioscorea* species were obtained from Eastern Asia, Southern North America and Southern Africa. Results showed the potential distribution of these *Dioscorea* species presented a higher degree of differentiation, and that new ecological suitability areas were mainly distributed in the central region of South America, the southern part of the European and coastal region of Oceania. The annual precipitation and annual mean radiation were the important climatic factors controlling the distribution of those *Dioscorea* species. The suitable areas and assessment of climatic factors will serve as a useful reference for the conservation, introduction and cultivation of diosgenin *Dioscorea* plants in ecological suitable areas.

## Additional files

**Additional file 1: Table S1.** Sample points and numbers of ten diosgenin-contained *Dioscorea* species. **Table S2.** Bioclimatic variables used as predictors in this study. **Table S3.** GenBank accessions of *matK* and *rbcL* sequences from *Dioscorea* and outgroup species. **Table S4.** Potential distribution sites and areas of diosgenin-contained *Dioscorea* species around the world (×10^5^ km^2^).

**Additional file 2.** Bioclimatic variables of 10 diosgenin-contained *Dioscorea* species.

**Additional file 3.** Minimum standards of reporting checklist.

**Additional file 4: Figure S1.** Boxplots are showing the percentage of stable habitat data of diosgenin-contained *Dioscorea* species under climate change models. **Figure S2.** Suitable areas for diosgenin-contained species from *Dioscorea.*
