# Supplementary material for: Predicting the potential global distribution of diosgenin-contained Dioscorea species
Source: Chin Med. 2018 Nov 19;13:58. doi: 10.1186/s13020-018-0215-8 (PMC6245757; doi:10.1186/s13020-018-0215-8)
Supplement: Supplementary file 1 — Additional file 1: Table S1. Sample points and numbers of ten diosgenin-contained Dioscorea species. Table S2. Bioclimatic variables used as predictors in this study. Table S3. GenBank accessions of matK and rbcL sequences from Dioscorea and outgroup species. Table S4. Potential distribution sites and areas of diosgenin-contained Dioscorea species around the world (×105 km2). [file 13020_2018_215_MOESM1_ESM.docx]

**Table S1** Sample points and numbers of ten diosgenin-contained *Dioscorea* species

| **Latin names** | **Sampling distribution** | **Sampling points** |
| --- | --- | --- |
| *D. althaeoides* | Mainly in China (Southwest part of Yunnan, Sichuan, Guizhou and Xizang; Central region of Shanxi, Chongqing and Hunan; Southern part of Zhejiang and Jiang). | 114 |
| *D. collettii* | Mainly in China (Sichuan, Yunnan, Guizhou, Chongqing, Guangxi, Hunan, Taiwan and Shanxi) and Honshu Japan. | 274 |
| *D. composita* | Mainly in southern part of Mexico (Tabasco, Veracruz, Oaxaca, [Chiapas](javascript:void(0);), Darfur's state and [Puebla](javascript:void(0);) etc; Eastern part of Guatemala (Cancuen, Livingston, Logo de lzobel, Morales); Central part of Belize; Yunnan pf China, Southern and eastern part of Burma, Northern and northwest part of India. | 190 |
| *D. deltoidea* | Mainly in Northern and central part of India，Sikkim, Bhutan, Nepal, Northeast part of Pakistan, few part of [Afghanistan](javascript:void(0);), Southwest part of China (Sichuan, Yunnan, Guizhou, Tibet and Taiwan), Southern part of Thailand and central region of Laos. | 108 |
| *D. gracillima* | Mainly in China (Zhejiang, Jiangxi, Anhui, Hunan and Fujian), Eastern part and [southeast](javascript:void(0);) of Japan (Tokyo, Yokohama, Nagano, Kyoto, [Fukuchiyama](javascript:void(0);), [Shimane](javascript:void(0);) and Kochi) and few part of Korea. | 176 |
| *D. nipponica* | Mainly in central, eastern, northern and [northeast](javascript:void(0);) part of China; Japan (Honshu, Shikoku and Kyushu); Eastern and southern part of Korea and Northern Korea; Far East region of Russia. | 212 |
| *D. panthaica* | Mainly in China (Sichuan, Yunnan, Guizhou, Chongqing, Hunan and Hubei) and northern part of Burma. | 115 |
| *D. spiculiflora* | Southern of Mexico (Yucatan, Triunfo, Tabasco, Yucatanae, Guerrero, Oaxaca, [Veracruz](javascript:void(0);), [Yucatan](javascript:void(0);), Tabasco etc), [Belize](javascript:void(0);) ([belmopan](javascript:void(0);), Chetumal etc); Guatemala (Guatemala, [Antigua](javascript:void(0);), Quezaltenango); Honduras (Tegucigalpa, Sanpedrosula, Catacamas); Nicaragua (Managua, Granada, Bluefields etc); Costa Rica (San Jose, Jaco, Cartago etc). | 186 |
| *D. sylvatica* | Mainly in eastern and northern part of South Africa, Central region and eastern part of [Zimbabwe](javascript:void(0);), Southeast part of Zambia, Central region of [Mozambique](javascript:void(0);). | 226 |
| *D. zingiberensis* | Shanxi, Sichuan, Hunan, Hubei, Chongqing, Yunnan, Jiangxi of China, Fukushima of Japan. | 207 |

**Table S2** Bioclimatic variables used as predictors in this study

| **Bioclimatic variable description** | **Abbreviation** | **Units** |
| --- | --- | --- |
| Annual mean temperature | T-aver | ℃ |
| Mean temperature of warmest quarter | T-warm | ℃ |
| Mean temperature of coldest quarter | T-cold | ℃ |
| Annual precipitation | Precipitation | mm |
| Annual radiation | Radiation | Lux |
| Annual relative humidity | Humidity | % |

**Table S3** GenBank accessions of *matK* and *rbcL* sequences from *Dioscorea* and outgroup species

| **Species** | ***matk*** | ***rbcL*** |
| --- | --- | --- |
| *D. althaeoides* | EU407548.1 | EU407550.1 |
| *D. collettii* | HQ637615.1 | HQ637766.1 |
| *D. composita* | KJ922812.1 | KU237201.1 |
| *D. deltoidea* | KX258659.1 | HQ637750.1 |
| *D. gracillima* | HQ637612.1 | HQ637763.1 |
| *D. nipponica* | HQ637578.1 | HQ637729.1 |
| *D. panthaica* | JQ259983.1 | JQ260131.1 |
| *D. spiculiflora* | JQ587399.1 | JQ591329.1 |
| *D. sylvatica* | KR086978.1 | AF307462.1 |
| *D. zingiberensi* | HQ637599.1 | HQ637743.1 |
| *Alisma plantago-aquatica* | KX526480.1 | L08759.1 |
| *Tacca chantieri* | JF956623.1 | JF944590.1 |

**Table S4** Potential distribution sites and areas of diosgenin-contained *Dioscorea* species around the world (×10^5^ km^2^)

| **Species** | **Distributions of Country** | **Areas** |
| --- | --- | --- |
| *D. deltoidea* | China, The U.S., Brazil, Australian, India, The republic of Congo, Kazakhstan, Mexico, Bolivia, South Africa, Argentina, Turkey etc. | 465.91 |
| *D. nipponica* | The U.S., China, Russia, Canada, Kazakhstan, Brazil, Ukraine, Spain, France, Burma, Japan, Angola etc. | 262.33 |
| *D.* *collettii* | Brazil, China, The U.S., The republic of Congo, Burma, Peru, Bolivia, Mexico, Thailand, Tanzania, Angola etc. | 226.23 |
| *D. composita* | Brazil, The Republic of Congo, The U.S., India, China, Mexico, Angola, Bolivia, Burma, Zambia, Argentina, Tanzania etc. | 205.24 |
| *D. spiculiflora* | Mexico, Brazil, Australian, Indonesia, Argentina, Tanzania, India, The U.S., Burma, Ethiopia, South Africa, Bolivia etc. | 158.16 |
| *D. sylvatica* | Australian, Argentina, South Africa, Mexico, Zambia, Botswana, Angola, The U.S., Zimbabwe, Ethiopia, Morocco, Tanzania etc. | 107.40 |
| *D. althaeoides* | China, The U.S., France, Russia, Italy, Ukraine, Brazil, Australian, Spain, Rumania, New Zealand, Bulgaria etc. | 68.37 |
| *D. zingiberensis* | China, The U.S., Brazil, France, Japan, Italy, Rumania, Russia, Spain, Bulgaria, Australian, Portugal etc. | 65.89 |
| *D. gracillima* | China, The U.S., Japan, Canada, France, Russia, Italy, Rumania, New Zealand, Spain, Bulgaria, Serbia etc. | 60.48 |
| *D. panthaica* | China, The U.S., France, Italy, Brazil, Spain, Rumania, Korea, New Zealand, Japan, Bulgaria, Portugal etc. | 59.02 |
