# Supplementary material for: Predicting the potential global distribution of diosgenin-contained Dioscorea species
Source: Chin Med. 2018 Nov 19;13:58. doi: 10.1186/s13020-018-0215-8 (PMC6245757; doi:10.1186/s13020-018-0215-8)
Supplement: Supplementary file 4 — Additional file 4: Figure S1. Boxplots are showing the percentage of stable habitat data of diosgenin-contained Dioscorea species under climate change models. Figure S2. Suitable areas for diosgenin-contained species from Dioscorea. [file 13020_2018_215_MOESM4_ESM.docx]

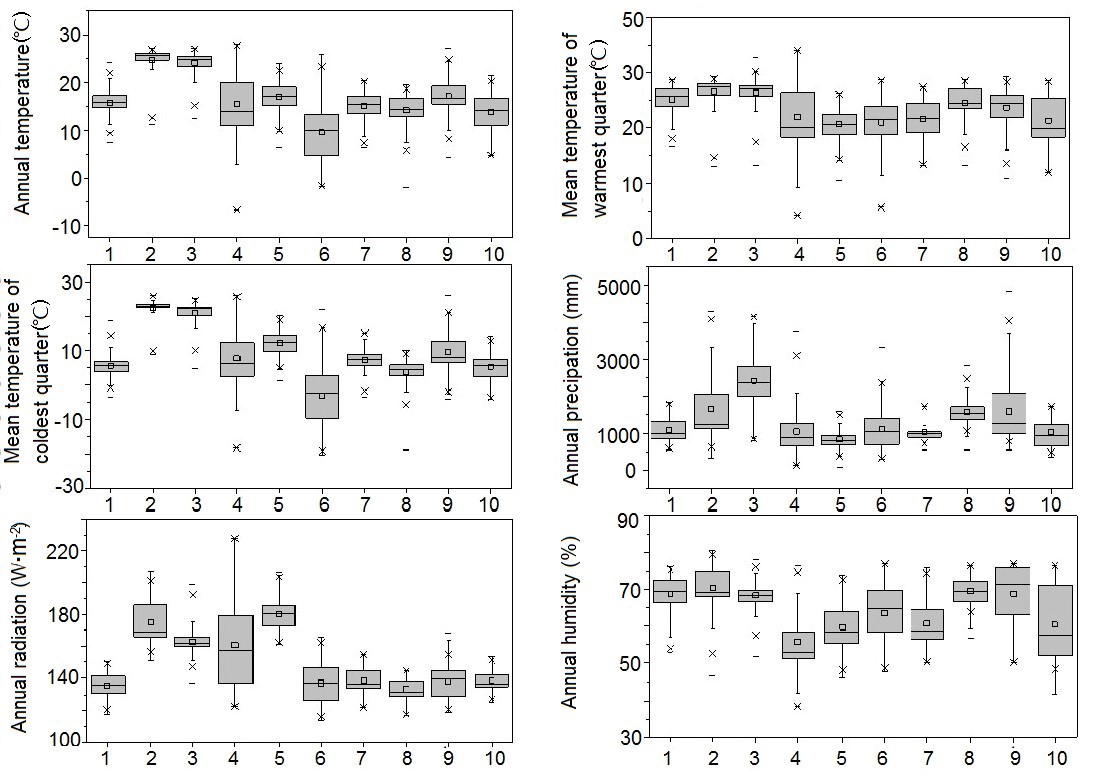


**Figure S1** Boxplots are showing the percentage of stable habitat data of diosgenin-contained *Dioscorea* species under climate change models. Boxplots are showing the distribution (mean, 25% and 75% percentiles, and extremes) of ten *Dioscorea* species. The proportion of total regional habitat data from samples points of *Dioscorea* species. Crosses indicate the proportion of total regional habitat remaining for the species. (1): *D. deltoidea*, (2): *D. nipponica*, (3): *D. collettii*, (4): *D. spiculiflora*, (5): *D. composita*, (6): *D. sylvatica*, (7): *D. zingiberensis*, (8): *D. panthaica*, (9): *D. althaeoides*, (10): *D. gracillima*.

| **A** | **B** |
| --- | --- |
| **C** | **D** |
| **E** | **F** |
| **G** | **H** |
| **I** | **J** |

**Figure S2** Suitable areas for diosgenin-contained species from *Dioscorea.* (**A**) *D. deltoidea*, (**B**) *D. nipponica*, (**C**) *D. collettii*, (**D**) *D. composita*, (**E**) *D. spiculiflora*, (**F**) *D. sylvatica*, (**G**) *D. althaeoides*, (**H**) *D. zingiberensis*, (**I**) *D. gracillima*, (**J**) *D. panthaica*
